# Evaluating new treatments for advanced cancer – reply

**DOI:** 10.1054/bjoc.2001.1890

**Published:** 2001-07

**Authors:** Jamie Ferguson

**Affiliations:** Public Health Medicine


doi: 10.1054/ bjoc.2001.1887, available online at http://www.idealibrary.com on IDEAL®

### Evaluating new treatments for advanced cancer

**New treatments for advanced cancer: an approach to prioritization**
**JSJ Ferguson, M Summerhayes, S Masters, S Schey and IE smith**
**Br J Cancer 83: 1268–1273**

**Sir**,
I would like to bring your attention to a number of significant errors in the above article which are extremely misleading, both in terms of the clinical effectiveness and cost of the drugs evaluated. For ease of reference I have listed where mistakes have been made below:

**Table 1** Gemcitabine is also licensed for treatment of advanced bladder cancer (muscle invasive Stage IV tumours with and without metastases) in combination with cisplatinum.

Paclitaxel is also licensed for the treatment of non small cell lung cancer (NSCLC)

Docetaxel is also licensed for the treatment of second line NSCLC.

The table, as it stands, is incomplete and misleading about the licensed indications of these drugs.

**Table 1** The costs have been calculated on a 'per cycle basis'.

The costs of Gemcitabine per cycle is listed as £1030, and the cost of Vinorelbine £175 – suggesting that Gemcitabine is approximately 6 times as expensive as Vinorelbine on a per cycle basis. The actual costs are in fact similar (for a *cycle* or course of treatment) and the table should therefore be corrected, so that *3 infusions* of Gemcitabine are not compared to *one infusion* of Vinorelbine.

**Table 3** The effectiveness of new treatment scale claims that Vinorelbine/Cisplatin has a 3–6 month survival advantage over Cisplatin in first line NSCLC, with strength of evidence alpha +. It would be interesting to see on what data this claim is based. There is no mention of the comparable trial of Gemcitabine/Cisplatin vs Cisplatin – in the first line setting, which should be included here as it showed a significant survival advantage ($P = 0.004$). Added to which 39% one year survival for the Gemcitabine/Cisplatin combination is not surpassed by any similar Vinorelbine/Cisplatin combinations.

It is also worth noting that in the only comparative trial where Gemcitabine/Cisplatin and Vinorelbine/Cisplatin have been compared (although admitedly not compared head to head) the Gemcitabine/Cisplatin arm appeared extremely favourable to the Vinorelbine/Cisplatin arm in terms of survival, where the Vinorelbine/Cisplatin arm was dropped at the interim analysis stage due to inferior efficacy.

Gemcitabine has no licence for the second-line treatment of NSCLC and this statement is therefore incorrect and should be removed, added to which there are no randomized data to support this effectiveness claim in the second line setting.

There are a number of tumour types where new treatments are available that have not been listed in the table such as pancreatic cancer, bladder cancer and glioblastoma multiforme.

These are the major inaccuracies.

It is also worth commenting that whilst survival is often considered the most important endpoint in clinical trials, tumour types which are notoriously chemoresistant such as NSCLC, pancreatic cancer, and renal cancer can be discriminated against by using a generic ranking scale which compares relatively chemosensitive disease such as ovarian cancer, with those where survival benefits may be hard to show, but where improvement in quality of life may be just as important.

**Dr Nicholas Botwood**
**Research Physician Lilly Oncology**
ENC – SPC,
Sandler,
Comelia doi: 10.1054/ bjoc.2001.1890, available online at http://www.idealibrary.com on IDEAL®

### Evaluating new treatments for advanced cancer – reply

**Sir**,
Neither the use of gemcitabine in bladder cancer nor the use of docetaxel in non small cell lung cancer NSCLC were licensed for these indications in the UK at the time of writing or preparing the manuscript. More importantly there had been no demand from clinicians locally to use these products in these indications. This also explains the omission of paclitaxel treatment for NSCLC (licensed Nov 1998). It should be noted that in our next meeting to appraise new anticancer agents we will be considering the use of gemcitabine plus cisplatin in bladder cancer.

### Cost per cycle

Price does not affect a rating a drug receives. We would concede that if used as a price comparison for different treatments Table 1 could be misleading. However it would be extremely unwise to do this for several reasons including the variability in pricing between institutions and over time depending on the level of discount and the variant regimens of the different drugs used.

There is a good reason why the vinorelbine price per week is given. At the time of writing (and currently) the dosage schedules used for this drug are more variable than most and include once weekly continuous treatment – with such a treatment, the appropriate cycle length is very hard to define.

### Relative effectiveness of vinorelbine

There are many trials of vinorelbine in NSCLC that could be considered when appraising its efficacy. Probably the two most relevant are the following. A large trial ($n = 612$) by Chevalier et al

© 2001 Cancer Research Campaign 

(1994) showed median survival to be 40 weeks for vinorelbine plus cisplatin and 32 weeks for vindesine and cisplatin. Wozniak et al (1998) in another large ($n$ = 432) phase III study, reported median survivals of 8 and 6 months for vinorelbine plus cisplatin and cisplatin alone, respectively. Thus, on the basis of these two large studies the survival benefit of adding vinorelbine to cisplatin appears to be about 2 months.

These two trials were high-quality randomized phase III trials so any conclusion we drew from them (even if incorrect) could be described as being based on alpha + evidence. Although a 2 months survival benefit is less than the 3 months that would automatically qualify it as a B for clinical effectiveness, it is also more than is required to qualify it as a C. It is in situations such as this where the consensus meetings described in the paper are invaluable. Attendees felt that the QoL impact of vinorelbine plus cisplatin were such as to merit a B rating. If treatments were rated by the inflexible application of rules then there would be no need for consensus meetings.

The study Dr Botwood cites (Sandler et al, 2000) in support of gemcitabine was only published in the *Journal of Clinical Oncology* in January 2000, 3 months after the original submission of our manuscript.

### Gemcitabine licence

The original wording of the gemcitabine Summary of Product Characteristics in NSCLC reads ‘for the palliative treatment of adult patients with locally advanced or metastatic NSCLC’. This has subsequently been modified to include an indication for first-line use with cisplatin. The original wording says nothing about line of treatment and is open to interpretation. We would interpret it as meaning that gemcitabine monotherapy can be used in NSCLC as first-line treatment or at any point in treatment where the prescriber thinks fit. We would concede his point that an error has crept in suggesting that the evidence for using gemcitabine second-line is much weaker than Table 3.

### Endpoints

Dr Botwood makes a useful point that there are multiple outcomes, which can be assessed when appraising cancer treatments. Our own recent discussions acknowledge this and if he wishes to promote discussion on this matter we think this would be most interesting.

**Dr Jamie Ferguson**
**Consultant in Public Health Medicine**

### REFERENCES

Le Chevalier T, Brisgand D, Douillard JY, Pujol JL, Alberola V, Monnier A, Rivere A, Lianes P, Chomy P and Cigolari S (1994) Randomized study of vinorelbine and cisplatin versus vindesine and cisplatin versus vinorelbine alone in advanced non-small cell lung cancer: results of a European multicenter trial including 612 patients. *J Clin Oncol* **12**: 360–367

Sandler AB, Nemunaitis J, Denham C, von Pawel J, Cormier Y, Gatzemeier U, Mattson K, Manegold C, Palmer MC, Gregor A, Nguyen B, Niyikiza C and Einhorn LH (2000) Phase III trial of gemcitabine plus cisplatin versus cisplatin alone in patients with locally advanced or metastic non-small-cell lung cancer. *J Clin Oncol* **18**: 122–130

Wozniak AJ, Crowley JJ, Balcerzak SP, Weiss GR, Spiridonidis CH, Baker LH, Albain KS, Kelly K, Taylor SA, Gandara DR and Livinston RB (1998) Randomized trial comparing cisplatin plus vinorelbine in the treatment of advanced non-small cell lung cancer: a Southwest Oncology Group study. J Clin Oncol 16: 2459–2465

### Editor’s comment

These letters raise a number of important issues for evaluating cancer therapies. New drugs or new combinations are being tested all the time and even in the time between writing and acceptance of the paper by Ferguson et al, there were important new trials that change the perspective on treatment of common cancers. At a time when national programmes of evaluation, such as those of the National Institute for Clinical Excellence (NICE), are increasingly being applied to new cancer drugs, it is important that the limitations of such analyses are recognized.

(1) The analyses need to be continually updated in the light of new data.
(2) The most appropriate tools for evaluating palliative treatments without significant survival benefit are not well established.

It is also important that the outcomes of such analyses are not applied too rigidly. In many instances the difference between comparable treatments is small and side effects may vary. In these circumstances, clinical judgement and patient choice may be important features in deciding the most appropriate treatment.

**Robert Hawkins**
**Clinical Editor British Journal of Cancer**
E-mail: RHawkins@picr.man.ac.uk
Fax: 01625–820368

 *© 2001 Cancer Research Campaign*

